# Construction of a predictive model for depressive symptoms following acute myocardial infarction and its impact on prognosis

**DOI:** 10.3389/fpsyt.2025.1431182

**Published:** 2025-04-29

**Authors:** Lei Ren, Hongqi Wang, Xin Su, Yangyang Yang, Yuanzhuo Zhang, Xiaoyan Yin, Dapeng Zhang, Guangquan Hu, Bin Ning

**Affiliations:** ^1^ Department of Cardiovascular Medicine, Fuyang People’s Hospital Affiliated to Anhui Medical University (Fuyang People’s Hospital), Fuyang, China; ^2^ Department of Cardiovascular Medicine, Fuyang Hospital Affiliated to Bengbu Medical College (Fuyang People’s Hospital), Fuyang, China; ^3^ Department of Psychiatry, Fuyang Third People’s Hospital, Fuyang, China; ^4^ Department of Cardiovascular Medicine, The Second Affiliated Hospital of Anhui Medical University, Hefei, China

**Keywords:** acute myocardial infarction, depressive symptoms, predictive model, prognosis, major adverse cardiovascular events

## Abstract

**Objective:**

To construct a predictive model for depressive symptoms following acute myocardial infarction (AMI) and analyze its impact on patient outcomes.

**Methods:**

A retrospective analysis was conducted on the clinical data of 216 patients who successfully underwent percutaneous coronary intervention (PCI) for AMI at the hospital from January 2022 to June 2023. One month post-PCI, patients were categorized into groups with and without depressive symptoms based on the Self-Rating Depression Scale (SDS) scores. Logistic regression was used to identify factors influencing depressive symptoms, and a nomogram model for post-PCI depressive symptoms risk in AMI patients was developed using these factors. Internal validation of the model was performed using the Bootstrap method and Hosmer-Lemeshow goodness-of-fit test. The model’s value was assessed through Receiver Operating Characteristic (ROC) curve analysis. Outcomes at six months post-PCI were also compared between patients with different levels of depressive symptoms.

**Results:**

At one month post-PCI, the incidence of depressive symptoms was 54.63%. Logistic regression revealed that Killip class III, monocyte count, albumin levels, C-reactive protein (CRP), and left ventricular ejection fraction (LVEF) were significant predictors of post-AMI depressive symptoms (P < 0.05). The nomogram, based on these five primary indicators, showed good concordance with acceptable and ideal curves (Hosmer-Lemeshow test χ2 = 10.593, P = 0.226); the area under the ROC curve was 0.767 (95% CI: 0.702-0.831). At six months post-PCI, the rates of rehospitalization and major adverse cardiovascular events were higher in the group with depressive symptoms compared to those without (P < 0.05); severe depressive symptoms were associated with a higher rate of major adverse cardiovascular events than mild depressive symptoms (P < 0.05).

**Conclusion:**

Killip class III, monocyte count, albumin levels, CRP, and LVEF are significant predictors of post-AMI depressive symptoms. The predictive model based on these factors demonstrates good calibration and discriminative ability; moreover, depressive symptoms adversely affect the prognosis of AMI patients, with more severe symptoms correlating with a higher incidence of major adverse cardiovascular events.

## 1 Introduction

Percutaneous coronary intervention (PCI), although effective in alleviating the clinical symptoms of acute myocardial infarction (AMI) patients, involves a complex psychological process preoperatively (e.g., intense pain from the disease, traumatic treatments, high medical costs), and postoperatively, patients often face significant comorbid stress, frequently manifesting as anxiety and depression (1, 2). Statistics indicate that 54.7% of PCI patients experience varying degrees of anxiety and depression postoperatively (3). Studies have shown that depression is a significant factor in the poor prognosis of AMI, possibly exceeding the impact of traditional risk factors (4, 5). Clinical studies on emotional interventions for PCI patients have also demonstrated that such interventions can beneficially impact outcomes (6). In recent years, with the advancement of psychocardiology, the depressive states of patients with cardiovascular diseases have garnered extensive attention (7, 8). However, most current clinical studies focus primarily on the relationship between demographic data (such as age, education level, smoking) and depression in AMI patients (9, 10), with limited research on other objective clinical indicators, thus lacking effective therapeutic targets. Building on previous studies, this research aims to analyze the impact of clinical laboratory test indicators on depressive symptoms in AMI patients and construct a predictive model to evaluate its effects on patient outcomes, further refining current research and providing insights for improving patient prognoses.

## 2 Materials and methods

### 2.1 General information

Clinical data from AMI patients treated at the hospital from January 2022 to June 2023 were retrospectively analyzed using the hospital’s electronic medical record system. Inclusion criteria: (1) Diagnosis of AMI conforming to relevant standards (11); (2) Patients who underwent successful direct PCI within 12 hours of symptom onset; (3) Patients capable of reading and understanding, able to complete the relevant questionnaire on their own or with medical staff assistance once stabilized; (4) Patients aged over 18 years. Exclusion criteria: (1) Patients with a history of long-term psychiatric medication use or chronic psychiatric conditions such as dementia, anxiety, or depression; (2) Patients with concomitant valvular heart disease, cardiomyopathy, severe arrhythmias, or other severe conditions; (3) Patients with severe liver or kidney failure or malignant tumors; (4) Patients with acute infections prior to PCI; (5) Patients who died within one month post-PCI; (6) Patients with Killip class IV; (7) Patients who experienced significant life events within one month post-PCI; (8) Patients with missing baseline data, laboratory test results, or six-month post-procedure follow-up data. Following these criteria, 216 patients’ clinical data were included in this study. All emergency PCI procedures were performed by highly experienced cardiovascular specialists qualified in interventional cardiology. The ethical review number is [2022]155.

### 2.2 Methods

#### 2.2.1 Depression symptoms assessment and grouping

By consulting the electronic medical record system, patients were categorized into groups with depressive symptoms and normal groups based on their depression symptoms assessment scores one month post-PCI. Depression symptoms was assessed using the Self-rating Depression Scale (SDS) (12–14), which comprises 20 items, each scored from 1 to 4. After the patient completed the assessment, medical staff summed the scores of all items to calculate the raw score. This raw score was then multiplied by 1.25 and rounded to the nearest whole number to derive the standardized score. Patients with a standardized score of ≥53 were classified as having depressive symptoms and were grouped into the depressive symptoms group, while those with a score <53 were considered without depressive symptoms and grouped into the normal group. The severity of depression was categorized as mild (53-62 points), moderate (63-72 points), and severe (>72 points). The SDS score test results of this study were mainly done by cardiovascular physicians and Dr. Zhang Dapeng, a psychiatrist from the Third People’s Hospital of Fuyang City.

#### 2.2.2 General data collection

Demographic and clinical data were collected for both groups through the electronic medical record system, including gender, age, body mass index (BMI), smoking history, alcohol consumption, histories of hypertension, diabetes, and coronary artery disease, time from onset to PCI, Killip class, culprit vessel, heart rate, systolic blood pressure, and diastolic blood pressure.

#### 2.2.3 Clinical laboratory indicator collection

Relevant laboratory test results at admission were compiled through the electronic medical record system to include indicators that might influence depressive symptoms. These included white blood cell count, neutrophil count, lymphocyte count, monocyte count, eosinophil count, red blood cell count, hemoglobin, platelet count, alanine aminotransferase, aspartate aminotransferase, albumin, blood urea nitrogen, creatinine, uric acid, troponin I, N-terminal pro-B-type natriuretic peptide (NT-proBNP), creatine kinase-MB (CK-MB), serum potassium, serum sodium, C-reactive protein (CRP), fasting plasma glucose (FPG), glycosylated hemoglobin (HbA1c), thyroid-stimulating hormone (TSH), free triiodothyronine (FT3), free thyroxine (FT4), total cholesterol, triglycerides, high-density lipoprotein cholesterol (HDL-C), low-density lipoprotein cholesterol (LDL-C), left ventricular ejection fraction (LVEF), and left ventricular internal diameter.

#### 2.2.4 Prognostic outcomes

At six months post-PCI, the rates of rehospitalization and major adverse cardiovascular events (including all-cause mortality, non-fatal myocardial infarction, and repeat revascularization) were documented using the electronic medical record system.

### 2.3 Statistical methods

Clinical data were analyzed using SPSS software version 27.0. Continuous variables were tested for normality using the Shapiro-Wilk test and normally distributed data were presented as $\bar{x}$ ± SD and compared using the t-test; skewed data were presented as medians [M (P25, P75)] and analyzed using the Mann-Whitney U test. Categorical data were expressed as percentages and numbers and analyzed using the chi-square test. Ordinal data were evaluated with the rank-sum test. Logistic regression was used to identify factors influencing post-PCI depressive symptoms in AMI patients. All independent risk factors were incorporated into a nomogram model, constructed using the “rms” package in R software. The model’s internal validity was assessed with the Bootstrap method (1000 resamples) and the Hosmer-Lemeshow goodness-of-fit test. The predictive value of the model was evaluated using the receiver operating characteristic (ROC) curve and the area under the curve (AUC). All tests were two-tailed with a significance level set at α = 0.05.

## 3 Results

### 3.1 Depression post-PCI in AMI patients

Of the 216 patients studied, 118 exhibited depressive symptoms one month post-PCI, accounting for 54.63% of the cohort (118/216). The breakdown of depression severity included 74 cases of mild depression, 28 cases of moderate depression, and 16 cases of severe depression.

### 3.2 Comparison of baseline data and laboratory indicators between groups

There were statistically significant differences between the two groups in terms of Killip class, monocyte count, albumin, CRP, LDL-C, and left ventricular ejection fraction (LVEF) (P < 0.05). No significant differences were observed in the other data between the groups (P > 0.05). See **Table 1**.

**Table 1:** Comparison of baseline data and relevant laboratory indicators between groups.

Category	Depressive Symptoms Group (n=118)	Non-Depressive Symptoms Group (n=98)	Statistical Value	*P*
Gender [n (%)]				
Male	96 (81.36)	82 (83.67)	*χ* ^2^ = 0.198	0.656
Female	22 (18.64)	16 (16.33)		
Age ( $\bar{x}$ ± SD, years)	59.83 ± 14.38	56.85 ± 10.54	*t*=1.756	0.081
BMI( $\bar{x}$ ± SD, kg/m^2^)	24.72 ± 3.57	25.06 ± 3.17	*t*=0.726	0.468
Smoking History [n (%)]				
Yes	69 (58.47)	57 (58.16)	*χ* ^2^ = 0.002	0.963
No	49 (41.53)	41 (41.84)		
History of Hypertension [n (%)]				
Yes	72 (61.02)	50 (51.02)	*χ* ^2^ = 2.177	0.140
No	46 (38.98)	48 (48.98)		
History of Diabetes [n (%)]				
Yes	40 (33.9)	24 (24.49)	*χ* ^2^ = 2.273	0.132
No	78 (66.1)	74 (75.51)		
History of Coronary Heart Disease [n (%)]				
Yes	89 (75.42)	66 (67.35)	*χ* ^2^ = 1.723	0.189
No	29 (24.58)	32 (32.65)		
Time from Onset to PCI( $\bar{x}$ ± SD, min)	68.32 ± 10.50	68.43 ± 10.61	*t*=0.074	0.941
Killip Class [n (%)]				
Class I	55 (46.61)	66 (67.35)	*Z*=3.151	0.002
Class II	22 (18.64)	14 (14.29)		
Class III	41 (34.75)	18 (18.37)		
Culprit Vessel [n (%)]				
Left Anterior Descending	47 (39.83)	49 (50.00)	*χ* ^2^ = 4.788	0.091
Right Coronary Artery	42 (35.59)	36 (36.73)		
Left Circumflex	29 (24.58)	13 (13.27)		
Heart Rates [*M*(*P*_25_, *P*_75_), Times/min]	75.00 (67.00, 88.25)	77.00 (66.00, 88.25)	*Z*=0.033	0.974
Systolic Blood Pressure [*M (P*_25_, *P*_75_), mmHg]	137.50 (117.75, 155.00)	133.50 (117.75, 150.00)	*Z*=0.561	0.575
Diastolic Blood Pressure [*M*(*P*_25_, *P*_75_), mmHg]	88.00 (73.00, 101.25)	86.00 (74.75, 98.00)	*Z*=0.280	0.779
White Blood Cells [*M*(*P*_25_, *P*_75_), ×10^9^/L]	7.66 (6.37, 9.30)	7.99 (6.19, 9.45)	*Z*=0.537	0.591
Neutrophil Count [*M*(*P*_25_, *P*_75_), ×10^9^/L]	5.19 (4.07, 6.58)	4.89 (4.00, 6.55)	*Z*=0.264	0.792
Lymphocyte Count [*M*(*P*_25_, *P*_75_), ×10^9^/L]	1.62 (1.28, 2.09)	1.59 (1.28, 2.17)	*Z*=0.124	0.902
Mononuclear Cell Count [*M*(*P*_25_, *P*_75_), ×10^9^/L]	0.55 ± 0.18	0.49 ± 0.17	*t*=2.641	0.009
Eosinophil Count [*M*(*P*_25_, *P*_75_), ×10^9^/L]	0.12 (0.06, 0.18)	0.11 (0.06, 0.19)	*Z*=0.416	0.678
Red blood cell count [[*M*(*P*_25_, *P*_75_), ×10^12^/L]	4.45 (4.05, 4.87)	4.46 (4.11, 4.82)	*Z*=0.769	0.442
Hemoglobin [*M*(*P*_25_, *P*_75_), g/L]	139.50 (124.00, 149.00)	138.50 (126.75, 151.25)	*Z*=0.807	0.420
Platelet count [*M*(*P*_25_, *P*_75_), ×10^9^/L]	203.00 (174.75, 259.00)	219.00 (181.50, 261.00)	*Z*=0.643	0.520
Alanine aminotransferase [*M*(*P*_25_, *P*_75_), U/L]	30.90 (21.45, 46.35)	28.80 (18.00, 46.05)	*Z*=1.529	0.126
Aspartate aminotransferase [*M*(*P*_25_, *P*_75_), U/L]	35.35 (25.65, 65.93)	29.95 (22.58, 55.58)	*Z*=1.473	0.141
Albumin [*M*(*P*_25_, *P*_75_), g/L]	37.45 (35.75, 40.75)	41.50 (38.48, 44.10)	*Z*=4.936	<0.001
Urea Nitrogen [*M*(*P*_25_, *P*_75_), mmol/L]	5.25 (4.18, 6.60)	5.35 (4.30, 6.60)	*Z*=0.486	0.627
Creatinine [*M*(*P*_25_, *P*_75_), μmol/L]	67.80 (60.13, 76.63)	66.70 (59.00, 76.93)	*Z*=1.020	0.308
Uric Acid [*M*(*P*_25_, *P*_75_), μmol/L]	318.94 ± 95.72	308.42 ± 99.28	*t*=0.791	0.430
Troponin I [*M*(*P*_25_, *P*_75_), ng/ml]	6.57 (2.50, 14.05)	8.51 (2.50, 28.64)	*Z*=1.153	0.249
NT pro-BNP [*M*(*P*_25_, *P*_75_), pg/ml]	625.00 (266.50, 1169.00)	552.00 (176.75, 974.25)	*Z*=1.276	0.202
CK-MB [*M*(*P*_25_, *P*_75_), ng/ml]	6.92 (2.89, 63.10)	9.60 (2.74, 47.71)	*Z*=0.031	0.976
Serum Potassium [*M*(*P*_25_, *P*_75_), mmol/L]	4.04 (3.79, 4.32)	3.99 (3.75, 4.20)	*Z*=1.548	0.122
Serum Sodium [*M*(*P*_25_, *P*_75_), mmol/L]	139.00 (137.00, 141.00)	138.71 (137.00, 140.11)	*Z*=0.754	0.451
CRP [*M*(*P*_25_, *P*_75_), mg/L]	9.24 (2.95, 29.76)	4.44 (2.42, 9.24)	*Z*=2.888	0.004
FPG [*M*(*P*_25_, *P*_75_), mmol/L]	6.10 (5.06, 8.05)	5.79 (5.25, 7.39)	*Z*=0.677	0.499
HbA1C [*M*(*P*_25_, *P*_75_), %]	6.20 (5.58, 7.40)	6.20 (5.70, 8.40)	*Z*=1.592	0.111
TSH [*M*(*P*_25_, *P*_75_), mIU/L]	0.90 (0.60, 1.77)	0.99 (0.61, 1.76)	*Z*=0.595	0.552
FT3 [*M*(*P*_25_, *P*_75_), pmol/L]	2.90 (2.64, 3.30)	2.86 (2.68, 3.22)	*Z*=0.201	0.841
FT4 [*M*(*P*_25_, *P*_75_), pmol/L]	1.20 (1.05, 1.35)	1.16 (1.03, 1.29)	*Z*=1.386	0.166
Total Cholesterol [*M*(*P*_25_, *P*_75_), mmol/L]	4.45 (3.72, 5.12)	4.57 (3.92, 5.33)	*Z*=1.082	0.279
Triglycerides [*M*(*P*_25_, *P*_75_), mmol/L]	1.42 (1.02, 2.04)	1.60 (1.03, 2.20)	*Z*=1.314	0.189
HDL-C [*M*(*P*_25_, *P*_75_), mmol/L]	0.92 (0.83, 1.16)	0.95 (0.82, 1.12)	*Z*=0.145	0.884
LDL-C [*M*(*P*_25_, *P*_75_), mmol/L]	2.35 (1.90, 3.07)	2.61 (2.23, 3.07)	*Z*=2.020	0.043
LVEF [*M*(*P*_25_, *P*_75_), %]	52.00 (46.00, 57.00)	56.50 (51.00, 62.00)	*Z*=3.553	<0.001
Left Ventricular Inner Diameter ( $\bar{x}$ ± s, mm)	48.05 ± 6.14	46.67 ± 5.98	*t*=1.660	0.098

### 3.3 Analysis of factors influencing depressive symptoms after PCI in AMI patients

Variables with statistical differences in **Table 1** were used as independent variables, with the occurrence of depressive symptoms after PCI in AMI patients as the dependent variable (1=depressed, 0=normal). Logistic regression analysis identified Killip class III, monocyte count, albumin, CRP, and LVEF as significant predictors of post-PCI depressive symptoms in AMI patients (P < 0.05). See **Table 2**.

**Table 2:** Analysis of factors influencing depressive symptoms after PCI in AMI patients.

Variable	*B*	*SE*	*WaIdχ* ^2^	*P*	*OR*	95% Confidence Interval
Killip Class	–	–	7.455	0.024	–	–
Class II	0.726	0.441	2.710	0.100	2.067	0.871-4.909
Class III	0.927	0.366	6.420	0.011	2.528	1.234-5.179
Monocyte Count	2.271	0.936	5.882	0.015	9.687	1.546-60.679
Albumin	-0.136	0.035	14.874	<0.001	0.873	0.815-0.935
CRP	0.020	0.009	4.825	0.028	1.020	1.002-1.039
LDL-C	-0.329	0.184	3.196	0.074	0.720	0.502-1.032
LVEF	-0.032	0.016	4.012	0.045	0.969	0.940-0.999
Constant	6.322	1.834	11.882	<0.001	–	–

### 3.4 Establishment and internal validation of the nomogram for predicting depressive symptoms risk after PCI in AMI patients

**Figure 1:**
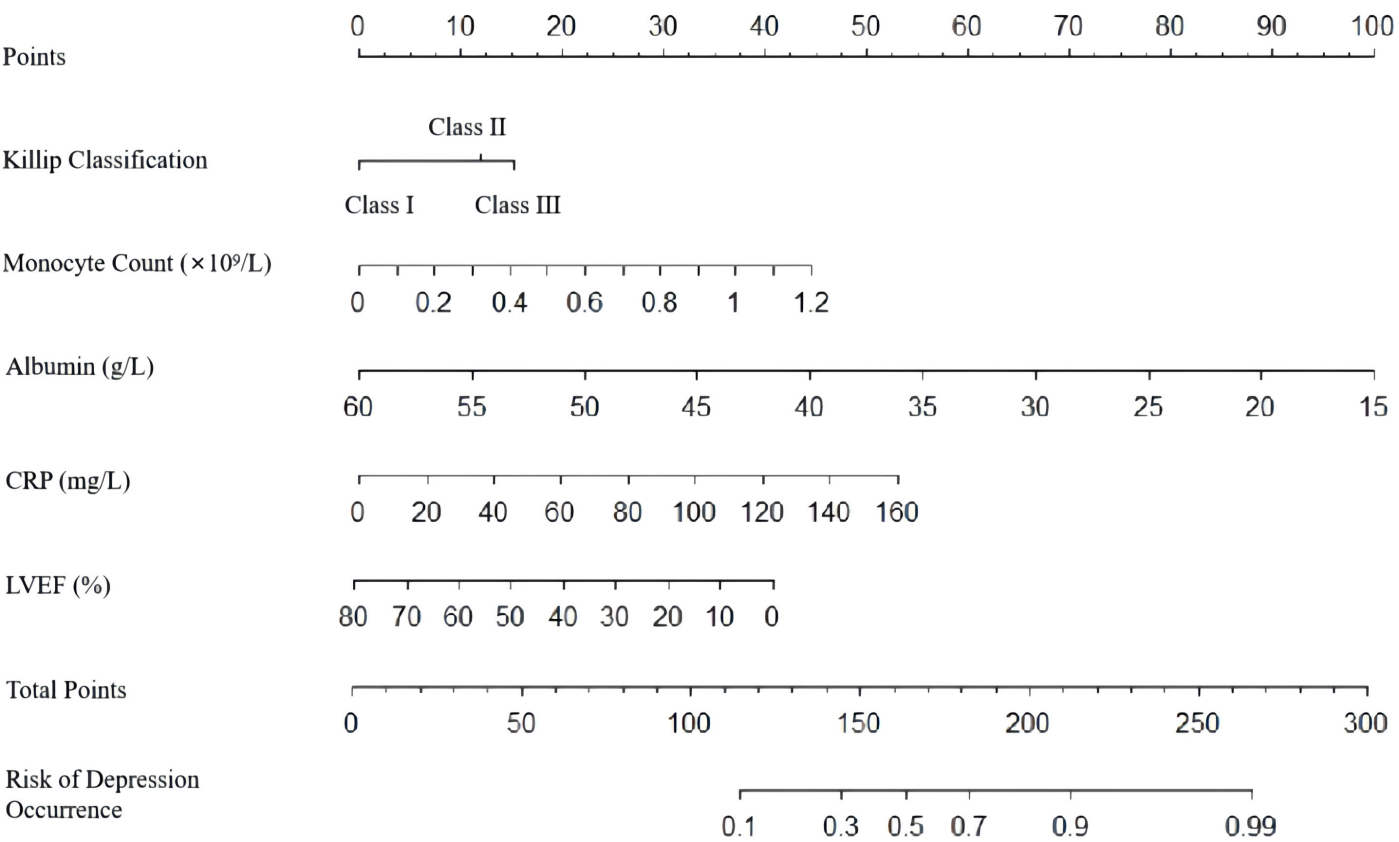
Nomogram for predicting depressive symptoms risk after PCI in AMI patients.

**Figure 2:**
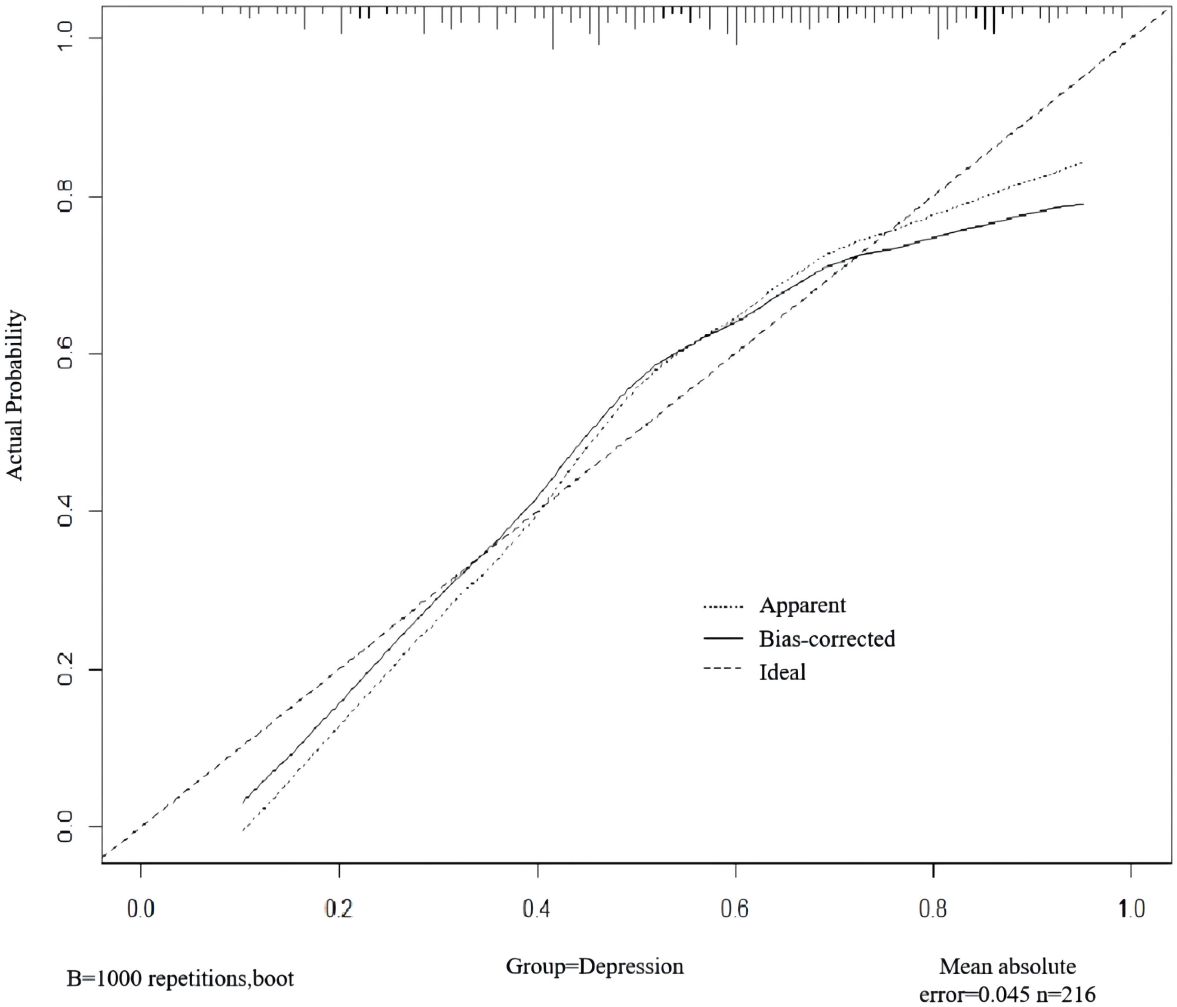
Internal validation of the nomogram for predicting depressive symptoms risk after PCI in AMI patients.

**Figure 3:**
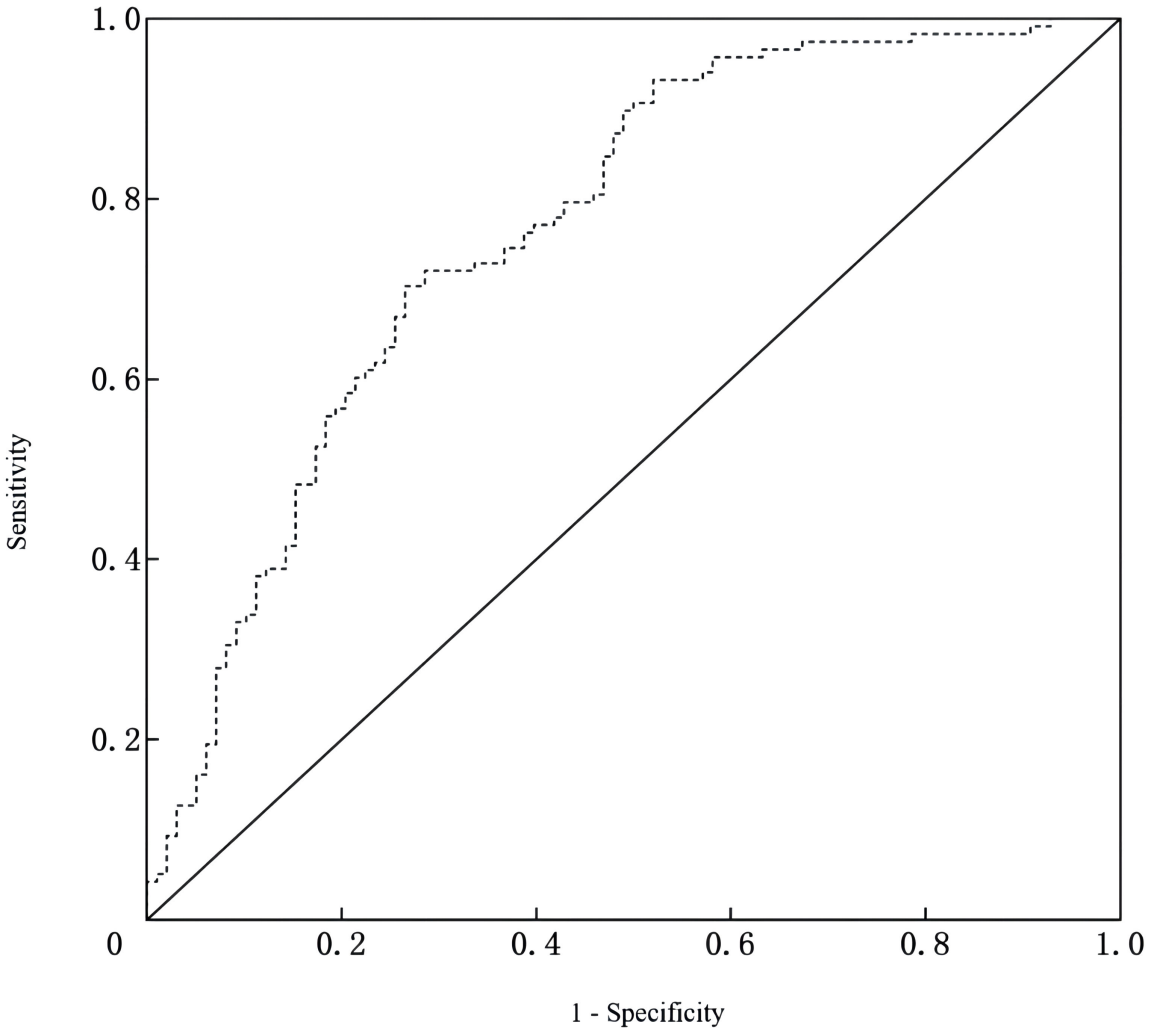
ROC curve of the nomogram for predicting depressive symptoms risk after PCI in AMI patients.

A nomogram was developed to predict the risk of depressive symptoms after PCI in AMI patients, as shown in **Figure 1**. The fit of the nomogram was confirmed with a Hosmer-Lemeshow test (*χ* ^2^ = 10.593, P = 0.226), indicating good calibration, as depicted in **Figure 2**. The receiver operating characteristic (ROC) curve demonstrated that the area under the curve (AUC) was 0.767 (95% CI: 0.702-0.831). At a cutoff value of 0.553, the model achieved high sensitivity (0.703) and specificity (0.735), with a Youden index of 0.438, as shown in **Figure 3**.

### 3.5 Prognostic outcomes

Six months post-PCI, the rates of rehospitalization and major adverse cardiovascular events were higher in the depressive symptoms group compared to the non-depressive symptoms group (P < 0.05). Patients with severe depression experienced a higher rate of major adverse cardiovascular events than those with mild depressive symptoms (P < 0.05). However, differences in rehospitalization rates among patients with varying degrees of depressive symptoms were not statistically significant (P > 0.05). See **Tables 3**, **4**.

**Table 3:** Comparison of prognostic outcomes between two groups (n (%)).

Group	Number	Rehospitalization Rate	Major Adverse Cardiovascular Events			
			Total Deaths	Nonfatal Myocardial Infarction	Revascularization	Total
Depressive Symptoms Group	118	21 (17.80)	1 (0.85)	8 (6.78)	11 (9.32)	19 (16.10)
Non-Depressive Symptoms Group	98	6 (6.12)	0	2 (2.04)	2 (2.04)	4( 4.08)
*χ* ^2^		6.671				12.060
*P*		0.010				<0.001

**Table 4:** Comparison of prognostic outcomes by different levels of depressive symptoms (n (%)).

Group	Number	Rehospitalization Rate	Major Adverse Cardiovascular Event Rate
Mild Depressive Symptoms	74	12 (16.22)	8 (10.81)
Moderate Depressive Symptoms	28	5 (17.86)	5 (17.86)
Severe Depressive Symptoms	16	4 (25.00)	6 (37.50)^a^
*χ* ^2^		0.688	6.961
*P*		0.709	0.031

Compared to patients with mild depressive symptoms, ^a^P<0.05.

## 4 Discussion

While PCI can save the lives of AMI patients, it can also lead to vascular restenosis post-PCI. Coupled with inadequate disease awareness, this often results in psychological disorders, among which depression is one of the most common post-PCI psychological disorders. Despite a decreasing trend over time, the incidence of postoperative depression in AMI patients remains significantly high. In our study, one month post-PCI, 54.63% of the 216 AMI patients exhibited depression, a rate consistent with findings by Miao et al. (3). Prior research has predominantly focused on the impact of demographic factors, personality, cognition, and social elements on depressive symptoms in AMI patients. However, our study emphasizes the influence of patients’ clinical conditions and laboratory indicators on the onset of depressive symptoms, thereby addressing some limitations of previous studies.

Clinically, our results indicate that the Killip class and LVEF values are significant factors affecting depressive symptoms post-PCI in AMI patients. Patients with a higher Killip class and lower LVEF values post-PCI exhibited a higher incidence of depressive symptoms. Current research on the impact of AMI patient conditions on depression varies greatly and lacks robust data support (15, 16). Nonetheless, studies have shown that psychocardiological interventions for AMI patients with comorbid anxiety and depression can significantly improve cardiac function indicators (17). Moreover, extensive research supports that cardiac rehabilitation can modulate the psychological state post-PCI in AMI patients (18, 19). Thus, Killip class and LVEF values can somewhat reflect the postoperative depression status of AMI patients. The rationale is that PCI facilitates revascularization and myocardial reperfusion, improving the patients’ quality of life and thereby reducing psychological stress, which in turn can decrease the occurrence and development of depressive states. However, patients with severe myocardial damage experience longer recovery times for cardiac function, leading to higher Killip classifications and lower LVEF values. The lack of immediate improvement in adverse symptoms may result in the persistence of negative emotions such as anxiety and depression. This suggests that clinicians should consider extending the follow-up period to monitor emotional changes in AMI patients after PCI, and implement postoperative strategies such as rehabilitation training and medication adherence to further promote cardiac function recovery and reduce the incidence of postoperative depression.

In terms of clinical laboratory indicators, this study demonstrates that albumin levels can influence the post-PCI depressive symptoms status in AMI patients. Studies have shown that malnutrition in elderly people can exacerbate depression, likely because depression might affect their appetite and reduce energy intake, thereby increasing their risk of malnutrition and affecting their psychological state (20, 21). Furthermore, a study in Ireland found significant correlations between nutritional indicators and symptoms of depression (22). Additionally, previous research has indicated that inflammatory responses play a role in the development of depression, suggesting that bodily inflammation can disrupt homeostasis and cause imbalances in neurotransmitter secretion (23, 24). Albumin, synthesized by the liver, reflects not only nutritional status but also the inflammatory state of the body, Pro-inflammatory factors can cross the blood-brain barrier, causing immune dysregulation in the central nervous system and leading to depression (25). A genome-wide association study revealed that genetic and epigenetic networks related to inflammation, such as polymorphisms in inflammatory mediators (CRP, tumor necrosis factor α, etc.), are closely associated with the severity of depression (26). Our study also found that CRP levels and monocyte counts are significant factors influencing depressive symptoms post-PCI in AMI patients, corroborating the aforementioned findings. Clearly, inflammatory cytokines may play a critical role in the pathophysiology of depression; thus, targeting inflammation through immunomodulation could provide insights for personalized treatment and drug development for depression (27, 28).

Furthermore, research has found that patients with depression exhibit multiple abnormalities in lipid profiles, which become more pronounced with the severity of depression, particularly in serum LDL-C levels. This may be related to inflammatory responses induced by abnormal LDL-C levels (29, 30). However, our study shows that serum LDL-C levels are not associated with depressive symptoms post-PCI in AMI patients; the specific reasons are unclear but may be related to differences in the study population and the relatively small sample size. Further in-depth analysis is required. Internal validation also revealed that the model has good calibration and discriminative ability, suggesting that this predictive model could aid clinical staff in the personalized identification of depression risk in AMI patients post-PCI.

Clinical research indicates that AMI patients with concomitant depression who undergo interventional treatment are more likely to experience myocardial reperfusion injury, subsequently increasing mortality rates (31). Negative emotions also serve as a risk factor for major adverse cardiovascular events (MACE) in patients with stable coronary artery disease (32). Studies on the related mechanisms have found that patients with anxiety or depression may exhibit elevated levels of inflammatory markers (such as CRP), altered platelet aggregability, and reduced sympathetic nerve activity, which can lead to an increased rate of MACE (33). Therefore, our study, observing patients six months post-PCI, shows that patients with depressive symptoms experienced significantly higher rates of MACE and rehospitalization compared to those without depressive symptoms. As the severity of depressive symptoms increased, so did the incidence of MACE, effectively validating the aforementioned mechanisms. However, regarding rehospitalization rates, differences between patients with varying degrees of depressive symptoms were minimal, likely due to the small sample sizes of patients with moderate and severe depression. Based on these findings, clinicians should conduct a detailed analysis of the relationship between the severity of depressive symptoms and rehospitalization rates to clarify the significance of this study.

In summary, Killip class III, monocyte count, albumin, CRP, and LVEF are significant factors influencing the occurrence of depressive symptoms post-PCI in AMI patients. Based on these factors, constructing a predictive model for the risk of depressive symptoms post-PCI in AMI patients has demonstrated high accuracy, aiding medical staff in the early identification of patients prone to depression. Moreover, depressive symptoms can adversely affect the prognosis post-PCI in AMI patients, with more severe symptoms correlating with higher rates of major adverse cardiovascular events. However, this study did not incorporate factors such as personality, cognition, and social elements at the time of data inclusion, and it lacks external validation in model verification. Therefore, future studies could conduct multicenter research, comprehensively include factors influencing depressive symptoms, continue to explore risk factors for depression post-PCI in AMI patients, enhance the predictive model, and perform external validations to improve the model’s generalizability.

## Data Availability

The original contributions presented in the study are included in the article/supplementary material. Further inquiries can be directed to the corresponding authors.

## References

[1] Saini RK Chaudhury S Singh N Chadha DS Kapoor R. Depression, anxiety, and quality of life after percuataneous coronary interventions. Ind Psychiatry J. (2022) 31:6–18. doi: 10.4103/ipj.ipj_126_21 35800859 PMC9255611

[2] Zhang Y Zhai Y Niu B Liu X Zhang X Wu S. Association between depression and clinical outcomes following percutaneous coronary intervention: A meta-analysis. Psychopathology. (2022) 55:251–7. doi: 10.1159/000524228 35421863

[3] Miao X Chen Y Qiu X Wang R. Construction and validation of a nomogram predicting depression risk in patients with acute coronary syndrome undergoing coronary stenting: A prospective cohort study. J Cardiovasc Dev Dis. (2023) 10:385. doi: 10.3390/jcdd10090385 37754813 PMC10532347

[4] Hou Y Zhang D Zhu J Zhao X Lu M Wu Q. Short report: depression and anxiety symptoms as predictors of adverse cardiovascular events in Chinese patients after percutaneous coronary intervention. Psychol Health Med. (2021) 26:1126–33. doi: 10.1080/13548506.2020.1837388 33073611

[5] Hou Y Zhao X Lu M Lei X Wu Q Wang X. Brief, one-on-one, telephone-adapted mindfulness-based stress reduction for patients undergoing percutaneous coronary intervention: a randomized controlled trial. Transl Behav Med. (2019) 9:1216–23. doi: 10.1093/tbm/ibz130 31504974

[6] Hu YY Jiang X Mao FY Zhang J Liu L Gu J. Effect of positive event recording based on positive psychology on healthy behaviors and readmission rate of patients after PCI: a study protocol for a prospective, randomized controlled trial. Trials. (2022) 23:1013. doi: 10.1186/s13063-022-06964-9 36514114 PMC9746175

[7] Ge Y Chao T Sun J Liu W Chen Y Wang C. Frontiers and hotspots evolution in psycho-cardiology: A bibliometric analysis from 2004 to 2022. Curr Probl Cardiol. (2022) 47:101361. doi: 10.1016/j.cpcardiol.2022.101361 35995242

[8] Ren L Li W Su X Yang Y Zhang Y Liu X. Follow-up study of depressive state on patients with atrial fibrillation 1 year after radio-frequency ablation. Front Psychiatry. (2022) 13:1046924. doi: 10.3389/fpsyt.2022.1046924 36620693 PMC9813399

[9] Khan Z Musa K Abumedian M Ibekwe M. Prevalence of depression in patients with post-acute coronary syndrome and the role of cardiac rehabilitation in reducing the risk of depression:A systematic review. Cureus. (2021) 13:e20851. doi: 10.7759/cureus.20851 35141096 PMC8802655

[10] Vulcănescu D Gheorman V Pîrvu DC Dinescu VC Gheorman V Udriștoiu I. Primary PCI and mental health: A 12-month follow-up study. Healthcare (Basel). (2023) 11:1620. doi: 10.3390/healthcare11111620 37297760 PMC10252455

[11] Thygesen K Alpert JS Jaffe AS Chaitman BR Bax JJ Morrow DA. Fourth universal definition of myocardial infarction (2018). Glob Heart. (2018) 13:305–38. doi: 10.1016/j.gheart.2018.08.004 30154043

[12] Dunstan DA Scott N. Clarification of the cut-off score for Zung’s self-rating depression scale. BMC Psychiatry. (2019) 19:177. doi: 10.1186/s12888-019-2161-0 31185948 PMC6558728

[13] Zung WW. A self-rating depression scale. Arch Gen Psychiatry. (1965) 12:63–70. doi: 10.1001/archpsyc.1965.01720310065008 14221692

[14] Quan D. Clinical validity of the self-assessment scale for anxiety and depression. Chin J Ment Health. (2012) 26:676–9. doi: 10.3969/j.issn.1000-6729.2012.09.007

[15] Bürker BS Hardersen RI Lappegård KT. Symptoms of depression, anxiety, and posttraumatic stress among patients with cardiac pacemakers. Int J Environ Res Public Health. (2022) 19:16838. doi: 10.3390/ijerph192416838 36554718 PMC9778963

[16] Zhang W Zhu G Li B Chen C Zhu Y. Effect of cardiac rehabilitation therapy on depressed patients with cardiac insufficiency after cardiac surgery. Open Med (Wars). (2023) 18:20230821. doi: 10.1515/med-2023-0821 38025544 PMC10656761

[17] Ren L Ning B Ma Z. A randomized controlled study of bicentric medical intervention in 86 acute myocardial infarction patients with comorbid anxiety and depression. Chin J Clin Psychol. (2021) 29:887–90. doi: 10.16128/j.cnki.1005-3611.2021.04.045

[18] Bermudez T Bierbauer W Scholz U Hermann M. Depression and anxiety in cardiac rehabilitation: differential associations with changes in exercise capacity and quality of life. Anxiety Stress Coping. (2022) 35:204–18. doi: 10.1080/10615806.2021.1952191 34269151

[19] Arjunan P Trichur RV. The impact of nurse-led cardiac rehabilitation on quality of life and biophysiological parameters in patients with heart failure: A randomized clinical trial. J Nurs Res. (2020) 29:e130. doi: 10.1097/JNR.0000000000000407 33031130 PMC7808349

[20] Pascual-Morena C Cavero-Redondo I Reina-Gutiérrez S Saz-Lara A López-Gil JF Martínez-Vizcaíno V. Prevalence of neuropsychiatric disorders in duchenne and becker muscular dystrophies: A systematic review and meta-analysis. Arch Phys Med Rehabil. (2022) 103:2444–53. doi: 10.1016/j.apmr.2022.05.015 35839922

[21] Pascual-Morena C Cavero-Redondo I Martínez-Vizcaíno V Sequí-Domínguez I Fernández-Bravo-Rodrigo J Jiménez-López E. Dystrophin genotype and risk of neuropsychiatric disorders in dystrophinopathies: A systematic review and meta-analysis. J Neuromuscul Dis. (2023) 10:159–72. doi: 10.3233/JND-221586 PMC10041431 36565132

[22] Huang Y Xu Y Liu A. Increased levels of serum glycosylated hemoglobin are associated with depressive symptoms in a population with cancer (≥49 years): an antidepressant-stratified analysis. Clin Interv Aging. (2021) 16:205–12. doi: 10.2147/CIA.S294704 PMC7866938 33564231

[23] Suneson K Lindahl J Chamli Hårsmar S Söderberg G Lindqvist D. Inflammatory depression-mechanisms and non-pharmacological interventions. Int J Mol Sci. (2021) 22:1640. doi: 10.3390/ijms22041640 33561973 PMC7915869

[24] McCormack C Abuaish S Monk C. Is there an inflammatory profile of perinatal depression. Curr Psychiatry Rep. (2023) 25:149–64. doi: 10.1007/s11920-023-01414-y 36947355

[25] Hughes MM Connor TJ Harkin A. Stress-related immune markers in depression: implications for treatment. Int J Neuropsychopharmacol. (2016) 19:pyw001. doi: 10.1093/ijnp/pyw001 26775294 PMC4926799

[26] Lynall ME Soskic B Hayhurst J Schwartzentruber J Levey DF Pathak GA. Genetic variants associated with psychiatric disorders are enriched at epigenetically active sites in lymphoid cells. Nat Commun. (2022) 13:6102. doi: 10.1038/s41467-022-33885-7 36243721 PMC9569335

[27] Beurel E Toups M Nemeroff CB. The bidirectional relationship of depression and inflammation: double trouble. Neuron. (2020) 107:234–56. doi: 10.1016/j.neuron.2020.06.002 PMC7381373 32553197

[28] Harsanyi S Kupcova I Danisovic L Klein M. Selected biomarkers of depression: what are the effects of cytokines and inflammation. Int J Mol Sci. (2022) 24:578. doi: 10.3390/ijms24010578 36614020 PMC9820159

[29] Tkachev A Stekolshchikova E Vanyushkina A Zhang H Morozova A Zozulya S. Lipid alteration signature in the blood plasma of individuals with schizophrenia, depression, and bipolar disorder. JAMA Psychiatry. (2023) 80:250–9. doi: 10.1001/jamapsychiatry.2022.4350 PMC9878436 36696101

[30] So HC Chau CK Cheng YY Sham PC. Causal relationships between blood lipids and depression phenotypes: a Mendelian randomisation analysis. Psychol Med. (2021) 51:2357–69. doi: 10.1017/S0033291720000951 32329708

[31] Harshfield EL Pennells L Schwartz JE Willeit P Kaptoge S Bell S. Association between depressive symptoms and incident cardiovascular diseases. JAMA. (2020) 324:2396–405. doi: 10.1001/jama.2020.23068 PMC7739139 33320224

[32] Shiga T. Depression and cardiovascular diseases. J Cardiol. (2023) 81:485–90. doi: 10.1016/j.jjcc.2022.11.010 36410589

[33] Li G Zhang L Liu M. Meta-analysis on inflammation and autonomic nervous system of coronary heart disease combined with depression. BMJ Open. (2024) 14:e079980. doi: 10.1136/bmjopen-2023-079980 PMC10921480 38453204

